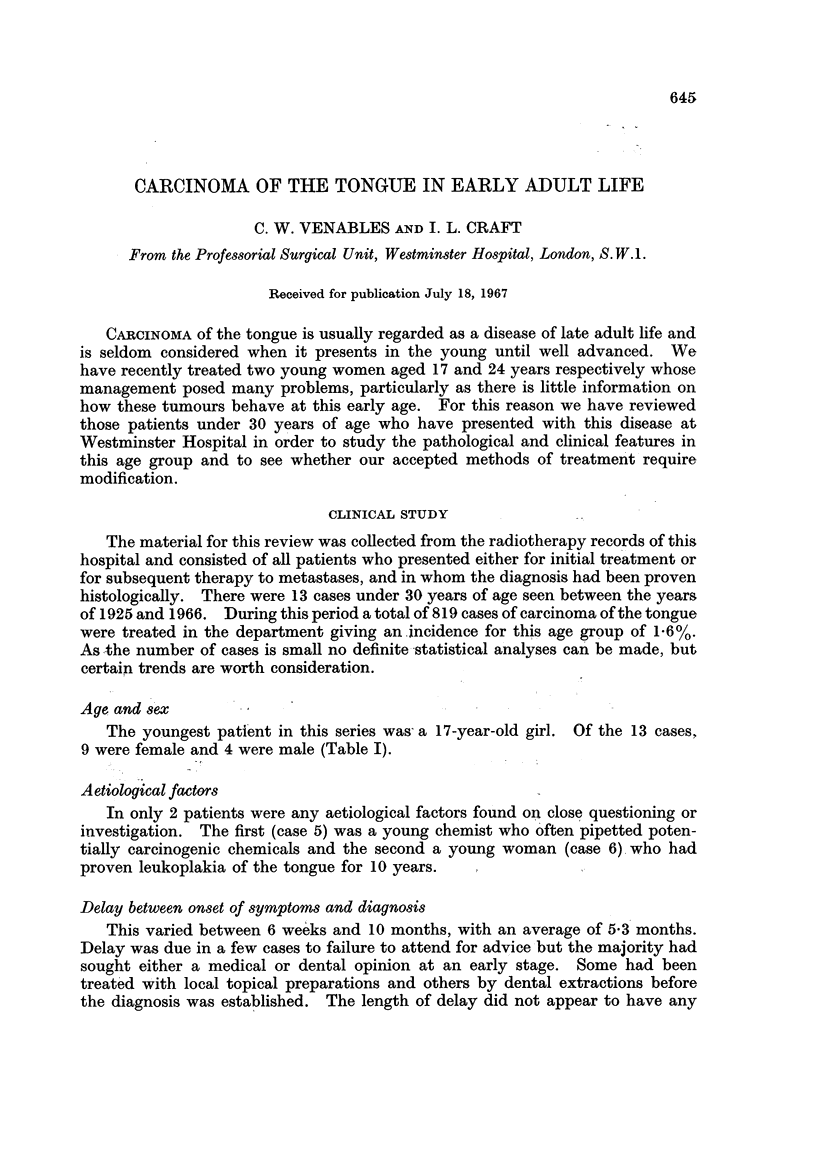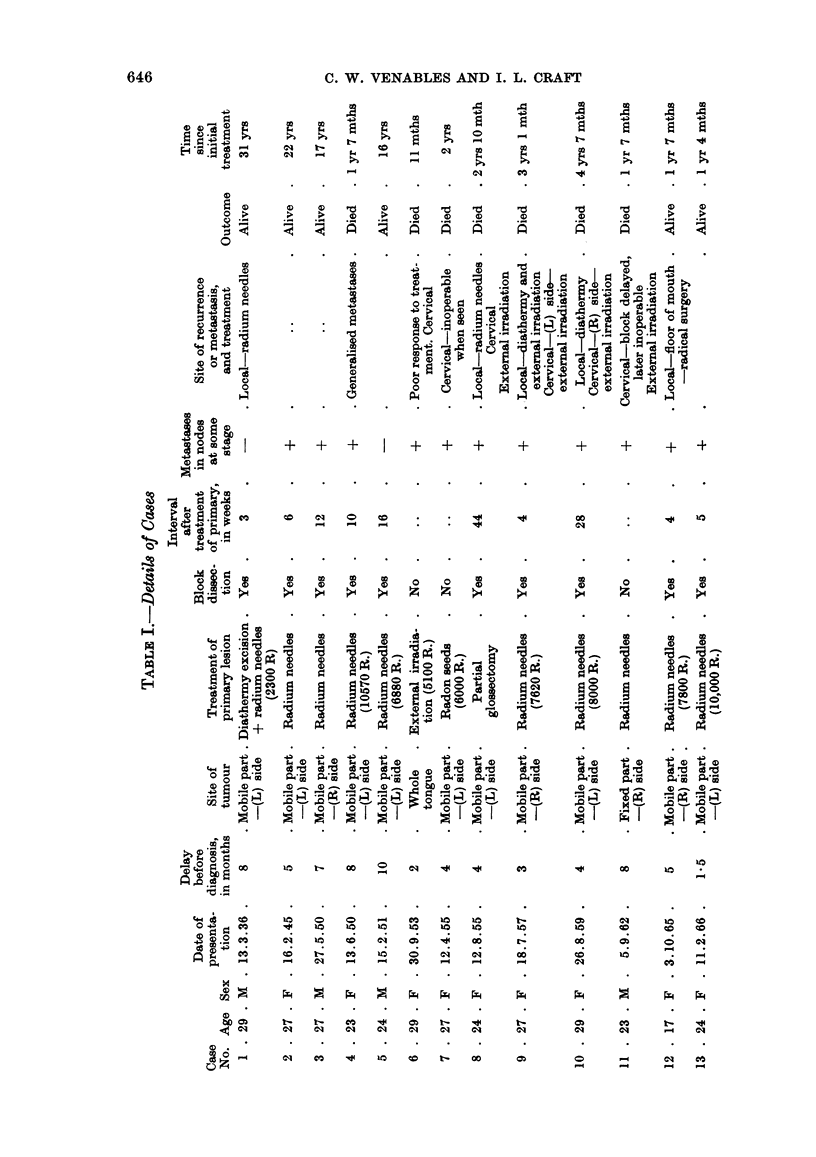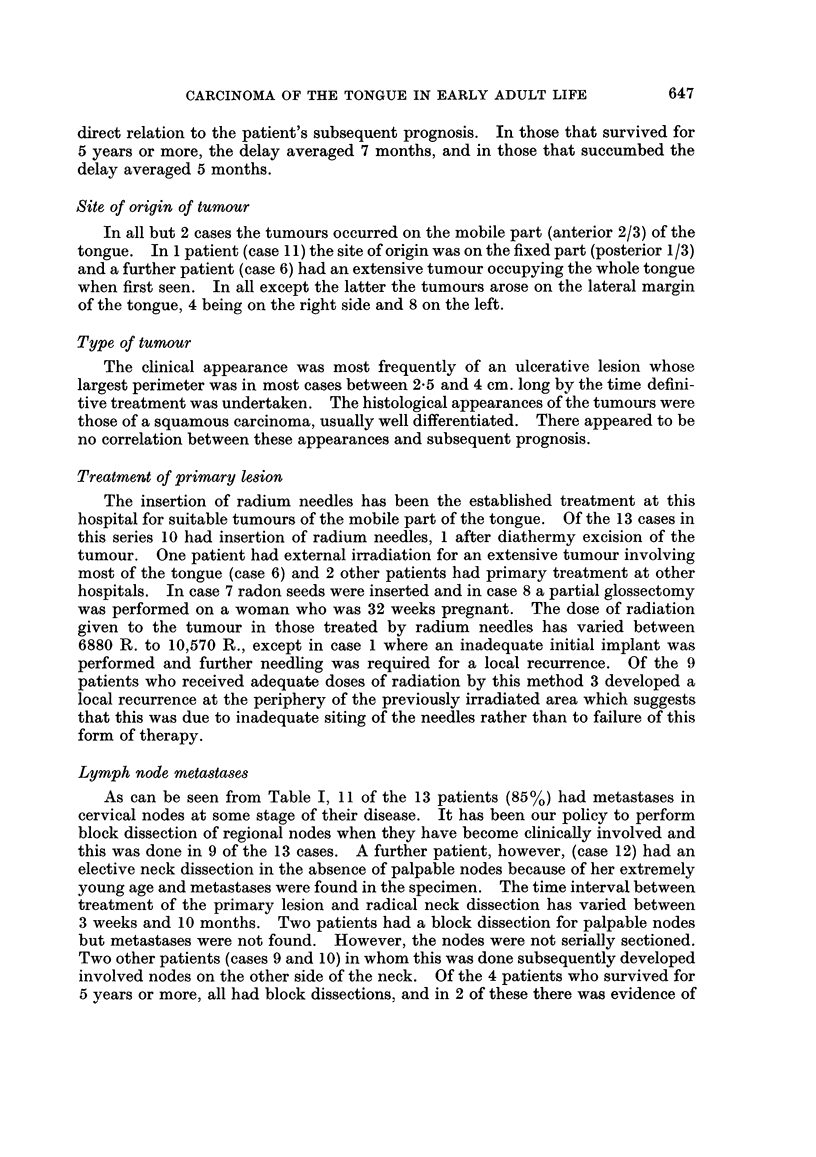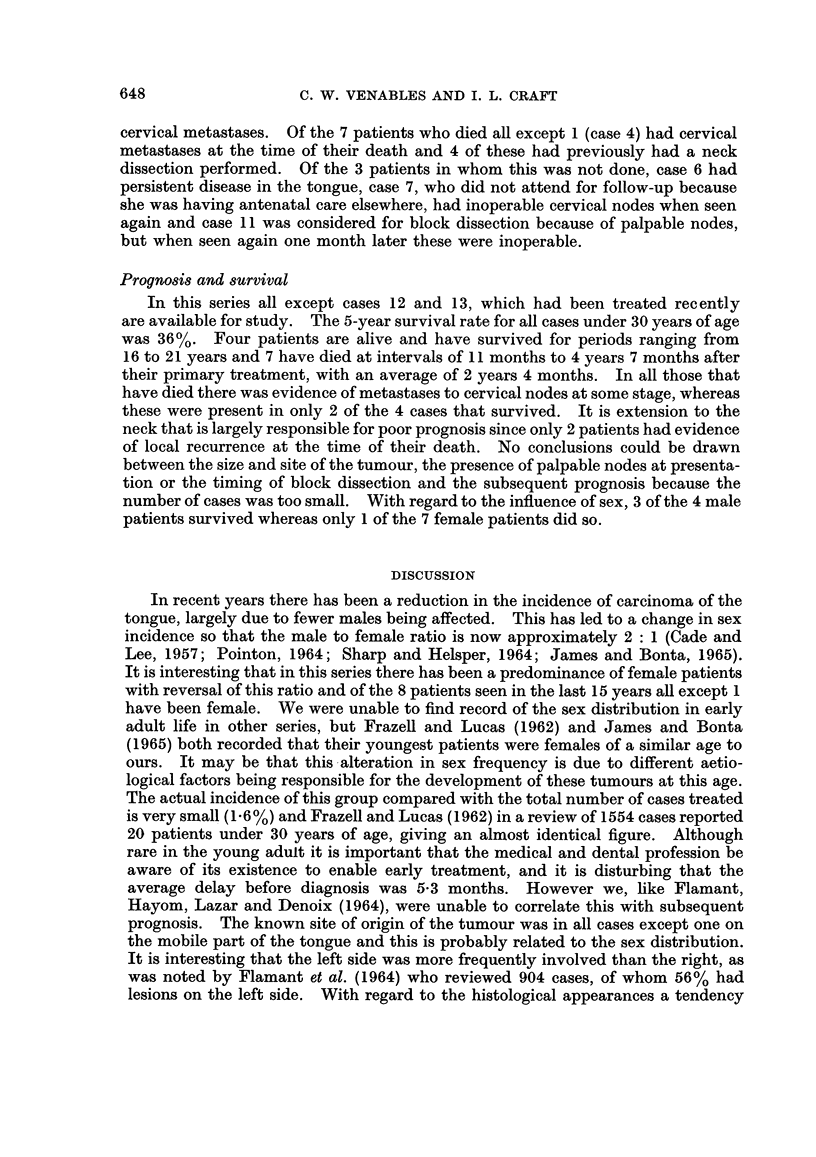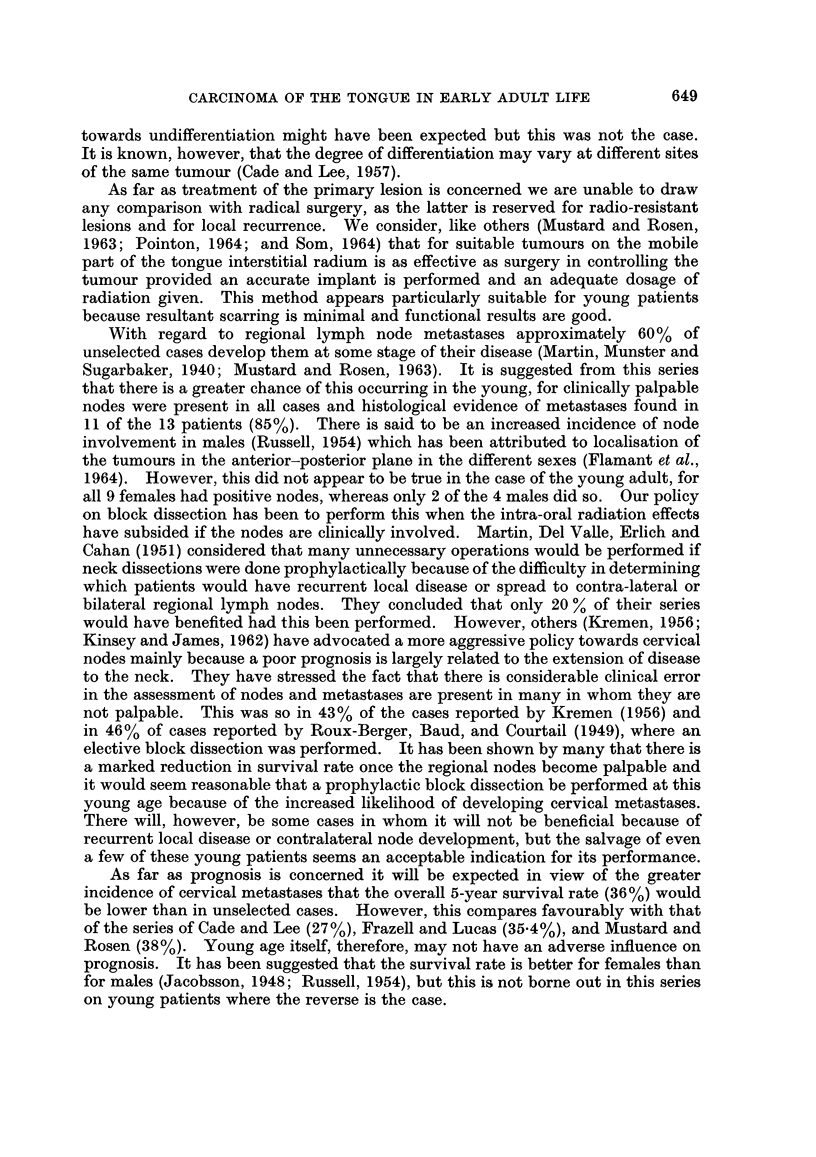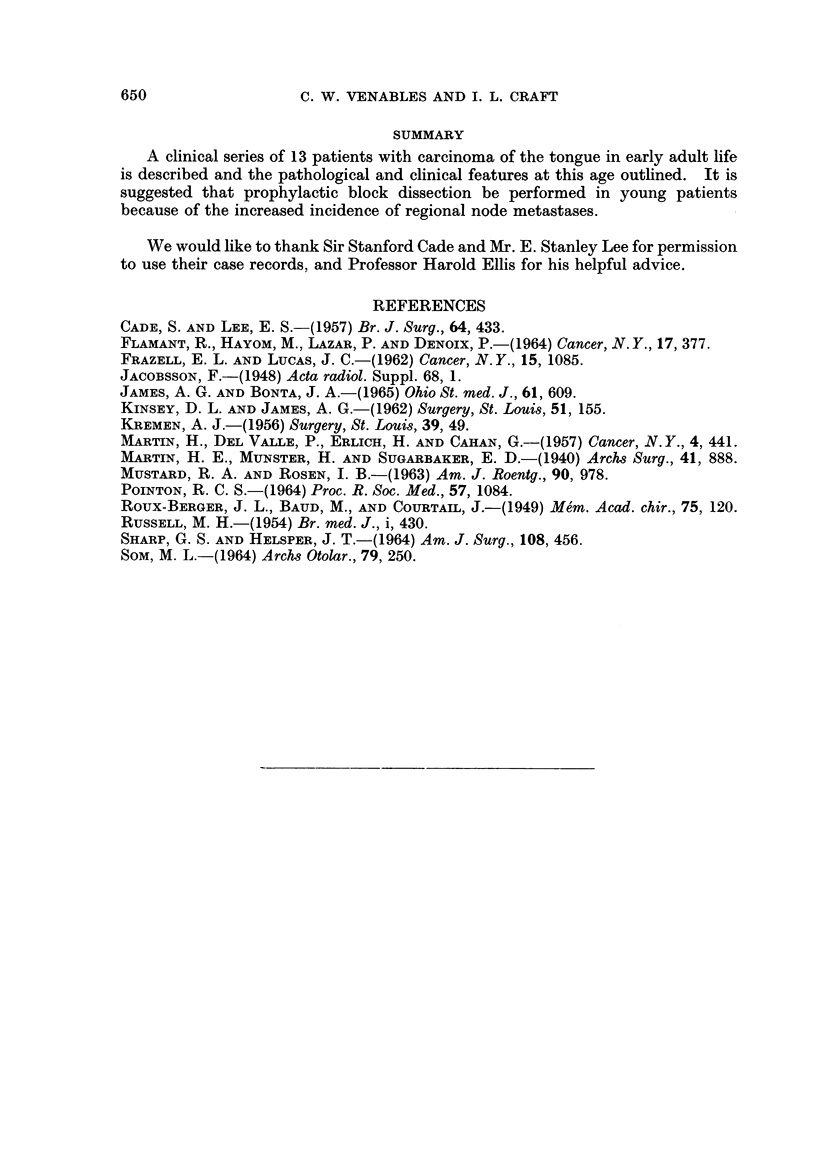# Carcinoma of the tongue in early adult life.

**DOI:** 10.1038/bjc.1967.75

**Published:** 1967-12

**Authors:** C. W. Venables, I. L. Craft


### CARCINOMA OF THE TONGUE IN EARLY ADULT LIFE

C. W. VENABLES AND I. L. CRAFT

*From the Professorial Surgical Unit, Westminster Hospital, London, S.W.1.*

Received for publication July 18, 1967

CARCINOMA of the tongue is usually regarded as a disease of late adult life and is seldom considered when it presents in the young until well advanced. We have recently treated two young women aged 17 and 24 years respectively whose management posed many problems, particularly as there is little information on how these tumours behave at this early age. For this reason we have reviewed those patients under 30 years of age who have presented with this disease at Westminster Hospital in order to study the pathological and clinical features in this age group and to see whether our accepted methods of treatment require modification.

### CLINICAL STUDY

The material for this review was collected from the radiotherapy records of this hospital and consisted of all patients who presented either for initial treatment or for subsequent therapy to metastases, and in whom the diagnosis had been proven histologically. There were 13 cases under 30 years of age seen between the years of 1925 and 1966. During this period a total of 819 cases of carcinoma of the tongue were treated in the department giving an incidence for this age group of 1·6%. As the number of cases is small no definite statistical analyses can be made, but certain trends are worth consideration.

### *Age and sex*

The youngest patient in this series was a 17-year-old girl. Of the 13 cases, 9 were female and 4 were male (Table I).

### *Aetiological factors*

In only 2 patients were any aetiological factors found on close questioning or investigation. The first (case 5) was a young chemist who often pipetted potentially carcinogenic chemicals and the second a young woman (case 6) who had proven leukoplakia of the tongue for 10 years.

### *Delay between onset of symptoms and diagnosis*

This varied between 6 weeks and 10 months, with an average of 5·3 months. Delay was due in a few cases to failure to attend for advice but the majority had sought either a medical or dental opinion at an early stage. Some had been treated with local topical preparations and others by dental extractions before the diagnosis was established. The length of delay did not appear to have any

TABLE I.—*Details of Cases*

| Case No. | Age | Sex | Date of presentation | Delay before diagnosis, in months | Site of tumour | Treatment of primary lesion | Block dissection | Interval after treatment of primary, in weeks | Metastases in nodes at some stage | Site of recurrence or metastasis, and treatment | Outcome | Time since initial treatment |
|---|---|---|---|---|---|---|---|---|---|---|---|---|
| 1 | 29 | M | 13.3.36 | 8 | Mobile part —(L) side | Diathermy excision + radium needles (2300 R) | Yes | 3 | — | Local—radium needles | Alive | 31 yrs |
| 2 | 27 | F | 16.2.45 | 5 | Mobile part —(L) side | Radium needles | Yes | 6 | + | .. | Alive | 22 yrs |
| 3 | 27 | M | 27.5.50 | 7 | Mobile part —(R) side | Radium needles | Yes | 12 | + | .. | Alive | 17 yrs |
| 4 | 23 | F | 13.6.50 | 8 | Mobile part —(L) side | Radium needles (10570 R.) | Yes | 10 | + | Generalised metastases | Died | 1 yr 7 mths |
| 5 | 24 | M | 15.2.51 | 10 | Mobile part —(L) side | Radium needles (6880 R.) | Yes | 16 | — | | Alive | 16 yrs |
| 6 | 29 | F | 30.9.53 | 2 | Whole tongue | External irradiation (5100 R.) | No | .. | + | Poor response to treatment. Cervical | Died | 11 mths |
| 7 | 27 | F | 12.4.55 | 4 | Mobile part —(L) side | Radon seeds (6000 R.) | No | .. | + | Cervical—inoperable when seen | Died | 2 yrs |
| 8 | 24 | F | 12.8.55 | 4 | Mobile part —(L) side | Partial glossectomy | Yes | 44 | + | Local—radium needles Cervical External irradiation | Died | 2 yrs 10 mth |
| 9 | 27 | F | 18.7.57 | 3 | Mobile part —(R) side | Radium needles (7620 R.) | Yes | 4 | + | Local—diathermy and external irradiation Cervical—(L) side—external irradiation | Died | 3 yrs 1 mth |
| 10 | 29 | F | 26.8.59 | 4 | Mobile part —(L) side | Radium needles (8000 R.) | Yes | 28 | + | Local—diathermy Cervical—(R) side—external irradiation | Died | 4 yrs 7 mths |
| 11 | 23 | M | 5.9.62 | 8 | Fixed part —(R) side | Radium needles | No | .. | + | Cervical—block delayed, later inoperable External irradiation | Died | 1 yr 7 mths |
| 12 | 17 | F | 3.10.65 | 5 | Mobile part —(R) side | Radium needles (7800 R.) | Yes | 4 | + | Local—floor of mouth —radical surgery | Alive | 1 yr 7 mths |
| 13 | 24 | F | 11.2.66 | 1·5 | Mobile part —(L) side | Radium needles (10,000 R.) | Yes | 5 | + | | Alive | 1 yr 4 mths |

direct relation to the patient's subsequent prognosis. In those that survived for 5 years or more, the delay averaged 7 months, and in those that succumbed the delay averaged 5 months.

*Site of origin of tumour*

In all but 2 cases the tumours occurred on the mobile part (anterior 2/3) of the tongue. In 1 patient (case 11) the site of origin was on the fixed part (posterior 1/3) and a further patient (case 6) had an extensive tumour occupying the whole tongue when first seen. In all except the latter the tumours arose on the lateral margin of the tongue, 4 being on the right side and 8 on the left.

*Type of tumour*

The clinical appearance was most frequently of an ulcerative lesion whose largest perimeter was in most cases between 2·5 and 4 cm. long by the time definitive treatment was undertaken. The histological appearances of the tumours were those of a squamous carcinoma, usually well differentiated. There appeared to be no correlation between these appearances and subsequent prognosis.

*Treatment of primary lesion*

The insertion of radium needles has been the established treatment at this hospital for suitable tumours of the mobile part of the tongue. Of the 13 cases in this series 10 had insertion of radium needles, 1 after diathermy excision of the tumour. One patient had external irradiation for an extensive tumour involving most of the tongue (case 6) and 2 other patients had primary treatment at other hospitals. In case 7 radon seeds were inserted and in case 8 a partial glossectomy was performed on a woman who was 32 weeks pregnant. The dose of radiation given to the tumour in those treated by radium needles has varied between 6880 R. to 10,570 R., except in case 1 where an inadequate initial implant was performed and further needling was required for a local recurrence. Of the 9 patients who received adequate doses of radiation by this method 3 developed a local recurrence at the periphery of the previously irradiated area which suggests that this was due to inadequate siting of the needles rather than to failure of this form of therapy.

*Lymph node metastases*

As can be seen from Table I, 11 of the 13 patients (85%) had metastases in cervical nodes at some stage of their disease. It has been our policy to perform block dissection of regional nodes when they have become clinically involved and this was done in 9 of the 13 cases. A further patient, however, (case 12) had an elective neck dissection in the absence of palpable nodes because of her extremely young age and metastases were found in the specimen. The time interval between treatment of the primary lesion and radical neck dissection has varied between 3 weeks and 10 months. Two patients had a block dissection for palpable nodes but metastases were not found. However, the nodes were not serially sectioned. Two other patients (cases 9 and 10) in whom this was done subsequently developed involved nodes on the other side of the neck. Of the 4 patients who survived for 5 years or more, all had block dissections, and in 2 of these there was evidence of cervical metastases. Of the 7 patients who died all except 1 (case 4) had cervical metastases at the time of their death and 4 of these had previously had a neck dissection performed. Of the 3 patients in whom this was not done, case 6 had persistent disease in the tongue, case 7, who did not attend for follow-up because she was having antenatal care elsewhere, had inoperable cervical nodes when seen again and case 11 was considered for block dissection because of palpable nodes, but when seen again one month later these were inoperable.

*Prognosis and survival*

In this series all except cases 12 and 13, which had been treated recently are available for study. The 5-year survival rate for all cases under 30 years of age was 36%. Four patients are alive and have survived for periods ranging from 16 to 21 years and 7 have died at intervals of 11 months to 4 years 7 months after their primary treatment, with an average of 2 years 4 months. In all those that have died there was evidence of metastases to cervical nodes at some stage, whereas these were present in only 2 of the 4 cases that survived. It is extension to the neck that is largely responsible for poor prognosis since only 2 patients had evidence of local recurrence at the time of their death. No conclusions could be drawn between the size and site of the tumour, the presence of palpable nodes at presentation or the timing of block dissection and the subsequent prognosis because the number of cases was too small. With regard to the influence of sex, 3 of the 4 male patients survived whereas only 1 of the 7 female patients did so.

### DISCUSSION

In recent years there has been a reduction in the incidence of carcinoma of the tongue, largely due to fewer males being affected. This has led to a change in sex incidence so that the male to female ratio is now approximately 2 : 1 (Cade and Lee, 1957; Pointon, 1964; Sharp and Helsper, 1964; James and Bonta, 1965). It is interesting that in this series there has been a predominance of female patients with reversal of this ratio and of the 8 patients seen in the last 15 years all except 1 have been female. We were unable to find record of the sex distribution in early adult life in other series, but Frazell and Lucas (1962) and James and Bonta (1965) both recorded that their youngest patients were females of a similar age to ours. It may be that this alteration in sex frequency is due to different aetiological factors being responsible for the development of these tumours at this age. The actual incidence of this group compared with the total number of cases treated is very small (1·6%) and Frazell and Lucas (1962) in a review of 1554 cases reported 20 patients under 30 years of age, giving an almost identical figure. Although rare in the young adult it is important that the medical and dental profession be aware of its existence to enable early treatment, and it is disturbing that the average delay before diagnosis was 5·3 months. However we, like Flamant, Hayom, Lazar and Denoix (1964), were unable to correlate this with subsequent prognosis. The known site of origin of the tumour was in all cases except one on the mobile part of the tongue and this is probably related to the sex distribution. It is interesting that the left side was more frequently involved than the right, as was noted by Flamant *et al.* (1964) who reviewed 904 cases, of whom 56% had lesions on the left side. With regard to the histological appearances a tendency towards undifferentiation might have been expected but this was not the case. It is known, however, that the degree of differentiation may vary at different sites of the same tumour (Cade and Lee, 1957).

As far as treatment of the primary lesion is concerned we are unable to draw any comparison with radical surgery, as the latter is reserved for radio-resistant lesions and for local recurrence. We consider, like others (Mustard and Rosen, 1963; Pointon, 1964; and Som, 1964) that for suitable tumours on the mobile part of the tongue interstitial radium is as effective as surgery in controlling the tumour provided an accurate implant is performed and an adequate dosage of radiation given. This method appears particularly suitable for young patients because resultant scarring is minimal and functional results are good.

With regard to regional lymph node metastases approximately 60% of unselected cases develop them at some stage of their disease (Martin, Munster and Sugarbaker, 1940; Mustard and Rosen, 1963). It is suggested from this series that there is a greater chance of this occurring in the young, for clinically palpable nodes were present in all cases and histological evidence of metastases found in 11 of the 13 patients (85%). There is said to be an increased incidence of node involvement in males (Russell, 1954) which has been attributed to localisation of the tumours in the anterior–posterior plane in the different sexes (Flamant *et al.*, 1964). However, this did not appear to be true in the case of the young adult, for all 9 females had positive nodes, whereas only 2 of the 4 males did so. Our policy on block dissection has been to perform this when the intra-oral radiation effects have subsided if the nodes are clinically involved. Martin, Del Valle, Erlich and Cahan (1951) considered that many unnecessary operations would be performed if neck dissections were done prophylactically because of the difficulty in determining which patients would have recurrent local disease or spread to contra-lateral or bilateral regional lymph nodes. They concluded that only 20 % of their series would have benefited had this been performed. However, others (Kremen, 1956; Kinsey and James, 1962) have advocated a more aggressive policy towards cervical nodes mainly because a poor prognosis is largely related to the extension of disease to the neck. They have stressed the fact that there is considerable clinical error in the assessment of nodes and metastases are present in many in whom they are not palpable. This was so in 43% of the cases reported by Kremen (1956) and in 46% of cases reported by Roux-Berger, Baud, and Courtail (1949), where an elective block dissection was performed. It has been shown by many that there is a marked reduction in survival rate once the regional nodes become palpable and it would seem reasonable that a prophylactic block dissection be performed at this young age because of the increased likelihood of developing cervical metastases. There will, however, be some cases in whom it will not be beneficial because of recurrent local disease or contralateral node development, but the salvage of even a few of these young patients seems an acceptable indication for its performance.

As far as prognosis is concerned it will be expected in view of the greater incidence of cervical metastases that the overall 5-year survival rate (36%) would be lower than in unselected cases. However, this compares favourably with that of the series of Cade and Lee (27%), Frazell and Lucas (35·4%), and Mustard and Rosen (38%). Young age itself, therefore, may not have an adverse influence on prognosis. It has been suggested that the survival rate is better for females than for males (Jacobsson, 1948; Russell, 1954), but this is not borne out in this series on young patients where the reverse is the case.

SUMMARY

A clinical series of 13 patients with carcinoma of the tongue in early adult life is described and the pathological and clinical features at this age outlined. It is suggested that prophylactic block dissection be performed in young patients because of the increased incidence of regional node metastases.

We would like to thank Sir Stanford Cade and Mr. E. Stanley Lee for permission to use their case records, and Professor Harold Ellis for his helpful advice.

### REFERENCES

Cade, S. and Lee, E. S.—(1957) *Br. J. Surg.*, **64**, 433.
Flamant, R., Hayom, M., Lazar, P. and Denoix, P.—(1964) *Cancer, N.Y.*, **17**, 377.
Frazell, E. L. and Lucas, J. C.—(1962) *Cancer, N.Y.*, **15**, 1085.
Jacobsson, F.—(1948) *Acta radiol.* Suppl. 68, 1.
James, A. G. and Bonta, J. A.—(1965) *Ohio St. med. J.*, **61**, 609.
Kinsey, D. L. and James, A. G.—(1962) *Surgery, St. Louis*, **51**, 155.
Kremen, A. J.—(1956) *Surgery, St. Louis*, **39**, 49.
Martin, H., Del Valle, P., Erlich, H. and Cahan, G.—(1957) *Cancer, N.Y.*, **4**, 441.
Martin, H. E., Munster, H. and Sugarbaker, E. D.—(1940) *Archs Surg.*, **41**, 888.
Mustard, R. A. and Rosen, I. B.—(1963) *Am. J. Roentg.*, **90**, 978.
Pointon, R. C. S.—(1964) *Proc. R. Soc. Med.*, **57**, 1084.
Roux-Berger, J. L., Baud, M., and Courtail, J.—(1949) *Mém. Acad. chir.*, **75**, 120.
Russell, M. H.—(1954) *Br. med. J.*, i, 430.
Sharp, G. S. and Helsper, J. T.—(1964) *Am. J. Surg.*, **108**, 456.
Som, M. L.—(1964) *Archs Otolar.*, **79**, 250.